# The association between dietary inflammation scores and non-alcoholic fatty liver diseases in Iranian adults

**DOI:** 10.1186/s12876-022-02353-3

**Published:** 2022-05-29

**Authors:** Hossein Farhadnejad, Asal Neshatbini Tehrani, Mitra Kazemi Jahromi, Farshad Teymoori, Ebrahim Mokhtari, Ammar Salehi-Sahlabadi, Parvin Mirmiran

**Affiliations:** 1 Student Research Committee, Nutrition and Endocrine Research Center, Research Institute for Endocrine Sciences, Shahid Beheshti University of Medical Sciences, Tehran, Iran; 2 Student Research Committee, Ahvaz Jundishapur University of Medical Sciences, Ahvaz, Iran; 3 Department of Nutrition, School of Allied Medical Sciences, Ahvaz Jundishapur University of Medical Sciences, Ahvaz, Iran; 4 Endocrinology and Metabolism Research Center, Hormozgan University of Medical Sciences, Bandar Abbas, Iran; 5 Nutrition and Endocrine Research Center, Research Institute for Endocrine Sciences, Shahid Beheshti University of Medical Sciences, Tehran, Iran; 6 Department of Nutrition, School of Public Health, Iran University of Medical Sciences, Tehran, Iran; 7 Department of Clinical Nutrition and Dietetics, Faculty of Nutrition and Food Technology, National Nutrition and Food Technology Research Institute, Shahid Beheshti University of Medical Sciences, Tehran, Iran

**Keywords:** Inflammation, Dietary pattern, Nonalcoholic fatty liver diseases, NAFLD, Adults

## Abstract

**Background:**

Potential dietary inflammation can precursor chronic diseases such as hepatic disorders. We aimed to examine the association of empirical dietary inflammatory patterns (EDIP) and dietary inflammation scores (DIS) with the risk of nonalcoholic fatty liver diseases (NAFLD) in Iranian adults.

**Methods:**

This case–control study was conducted on 225 newly diagnosed NAFLD cases and 450 controls aged 20–60 years. The individuals’ dietary data were collected using a validated food frequency questionnaire. The detection of NAFLD in subjects was done using the ultrasonography scan of the liver and confirmation of gastroenterologists. To calculate of EDIP score, the average daily intakes of each item (15 food items) were multiplied by the proposed weights, and then all the weighted values were summed. Also, to calculate the DIS score, each food item (18 food items) is multiplied by its specific weight to obtain the weighted values of each item. The weighted values were then standardized using the Z-score. Finally, the standardized weighted values of all the items were summed to get the overall DIS score for the individuals. Logistic regression models, adjusted for potential confounders, were used to estimate the odds ratios and 95% confidence interval (CI) of NAFLD across tertiles of EDIP and DIS.

**Results:**

The mean (SD) age and BMI of the study population (53% male) were 38.1 (8.8) years and 26.8 (4.3) kg/m^2^, respectively. The median (IQR) of EDIP and DIS scores in individuals were 0.52 (0.34, 0.73), and 0.04 (− 0.55, 0.59), respectively. Based on the multivariable-adjusted model, after controlling for age, sex, physical activity, smoking, marital status, waist-to-hip ratio, and dietary energy intake, individuals in the second (OR 2.01, 95% CI 1.07–3.76) and third tertiles of DIS (OR 2.54, 95% CI 1.39–4.63) had a higher odds of NAFLD compared to the lowest tertile of DIS (P_trend_ = 0.003). Also, in the final model, there is a significant direct association between EDIP score and odds of NAFLD [(OR T2 vs. T1 = 0.88, 95% CI 0.50–1.57) and (OR T3 vs. T1 = 1.82, 95% CI 1.02–3.23)], (P_trend_ = 0.031).

**Conclusion:**

Our results suggested that higher scores of EDIP and DIS, indicating the high inflammatory potential of dietary pattern, are associated with increased odds of NAFLD in Iranian adults.

## Background

Non-alcoholic fatty liver disease (NAFLD) is a major health problem that results from lipid accumulation when there is no other cause for liver damage, such as alcohol consumption and hepatitis viruses [1, 2]. NAFLD has several subtypes ranging from simple steatosis to non-alcoholic steatohepatitis (NASH) and finally to cirrhosis and hepatocellular carcinoma [3]. According to an estimation, around 20–30% of the general population suffers from NAFLD [2]. There is also a high prevalence of NAFLD in the Iranian people as a developing country [4]. Several risk factors attributed to NAFLD include dyslipidemia, type 2 diabetes (T2D), and other constituents of metabolic syndrome [5]. Among these, increasing body mass index (BMI) and visceral fat (%) are important risk factors in NAFLD incidence that two-thirds of people with obesity and diabetes are diagnosed with hepatic steatosis [6]. NAFLD has multifactorial pathogenesis encompassing environmental factors such as poor dietary choices and sedentary lifestyle along with genetic predisposition [7, 8].

Elevated systemic inflammation level can increase the risk of chronic diseases such as NAFLD [9–11]. Indeed, higher chronic inflammation characterized by higher inflammatory factors levels such as C-reactive protein (CRP) and interleukins (ILs) probably results from dietary and lifestyle exposures [12–15], increasing the risk of chronic illnesses such as NAFLD and premature death [13, 16, 17]. In this regard, previously, a pre-defined dietary inflammation index named dietary inflammatory index (DII) has been designed to examine the contribution of dietary exposures to systemic inflammation and, consequently, increase the chronic diseases risk such as NAFLD [18, 19]. Two studies showed that a diet with a higher DII score was associated with a higher degree of liver damage [19] and an increased risk of NAFLD [18].

Also, recently, the Byrd et al. study had developed two novel dietary inflammatory indices, including dietary inflammation score (DIS) [20] and empirical dietary inflammatory pattern (EDIP) [21], to assess the potential pro or anti-inflammatory effect of dietary pattern and the collective contributions of these dietary scores to systemic inflammation. Although several studies are available on the possible role of DIS and EDIP scores in increasing the risk of chronic diseases such as cancers, diabetes, and mortality [22–24], limited studies are conducted regarding the association between these novels' dietary inflammatory indices and the risk of NAFLD [25, 26]. A prospective cohort study reported that a dietary pattern with high inflammatory potential could be related to increased NAFLD risk [25]. Also, a nested case–control study reported that higher balances of pro- relative to anti-inflammatory dietary exposure, determined by DIS, may be linked with higher odds of metabolic-associated fatty liver disease [26].

Since it is now well established that individuals' dietary pattern has a remarkable role in determining the levels of systemic inflammation in the body and then inflammation-related diseases such as NAFLD and chronic liver ailments, thus the present study was conducted to investigate the association of the potential pro-inflammatory effect of dietary pattern, determined by two novels indices including DIS and EDIP, with risk of NAFLD.

## Materials and methods

### Study population

The present case–control study was conducted in the Metabolic Liver Disease Research Center as a referral center affiliated to Isfahan University of Medical Sciences on 225 newly diagnosed NAFLD patients and 450 controls aged 20–60 years. The diagnosis of NAFLD was confirmed by an ultrasound scan of the liver (grade II, III) in individuals without alcohol consumption and other causes of liver disease who were referred to screen for their probability of NAFLD due to abnormal liver enzymes levels or being at risk of metabolic syndrome, etc. The control group was selected based on liver ultrasound from individuals who had no stage of liver steatosis. Participants were included in the current study if they had no history of renal and hepatic diseases (Wilson’s disease, autoimmune liver disease, hemochromatosis, virus infection, and alcoholic fatty liver), CVD, diabetes, malignancy, thyroid disorder, and autoimmune history, were not on a special diet (due to a particular disease or weight loss) and do not use potentially hepatotoxic or steatogenic drugs. Participants who completed less than 35 items of the food frequency questionnaire (FFQ) and those with under or over-reported daily energy intake (≤ 800 or ≥ 4500 kcal/d) were excluded.

### Dietary assessment

The current study assessed dietary intakes using a validated and reproducible 168-item semi-quantitative food frequency questionnaire (FFQ) [27]. A list of typical Iranian foods with standard serving sizes was included in our FFQ. Participants were asked to report their average dietary intake during the previous year by choosing one of the following categories: never or less than once a month, 3–4 times per month, once a week, 2–4 times per week, 5–6 times per week, once daily, 2–3 times per day, 4–5 times per day, and six or more times a day. Portion sizes of each food item were converted into grams using standard Iranian household measures. The daily energy and nutrient intakes were computed based on the United States Department of Agriculture’s (USDA) Food Composition Table (FCT) [28]. For some local foods that are not available in USDA FCT, we used the Iranian FCT [29]. Then the food consumption frequencies were transformed into daily intakes.

### Calculation of indices

The DIS score was recently proposed by Byrd D.A et al. (30), basically based on 19 food items. Response variables for the development of this dietary index were interleukin-6, interleukin-8, interleukin-10, and high-sensitivity C-reactive protein (hs-CRP). Based on the effect of each item on the levels of inflammatory biomarkers, each item is assigned a specific weight that can be positive or negative. Because we have no information about supplement intake, we included 18 food groups in calculating the overall DIS score, including leafy greens and cruciferous vegetables, tomatoes, apples and berries, deep yellow or orange vegetables and fruit, other fruits, and natural fruit juices, other vegetables, legumes, fish, poultry, red and organ meats, processed meats, added sugars, high-fat dairy, low-fat dairy and tea, nuts, other fats, refined grains, and starchy vegetables. To calculate the DIS score, each food item is multiplied by its specific weight (introduced in Byrd et al. study [20]) to obtain the weighted values of each item. The weighted values were then standardized using the Z-score (to a mean of zero and SD of 1). Finally, the standardized weighted values of all the items were summed to get the overall DIS score for the individuals.

The EDIP score was calculated according to the Tabung et al. study [21], consisting of 18 food items. The diagnostic biomarkers used to construct this dietary index were interleukin-6, hs-CRP, and tumor necrosis factor-alpha (TNF-α). Each item's specific weight is assigned based on its relationship to the biomarker levels. Because alcoholic drinks such as wine and beer are not common or may be unreported in our study population due to religious considerations, we did not include them in calculating scores. Besides, since we have no food items as low-energy beverages in our FFQ, this item was excluded, too. Therefore we computed the EDIP score based on 15 instead of 18, including processed meat (sausage), red meat (beef, or lamb), organ meat (beef, calf, or chicken liver), other fish (canned tuna, or fish), other vegetables (mixed vegetables, green pepper, cooked mushroom, eggplant, zucchini, or cucumber), refined grains (white bread, biscuit, white rice, pasta, or vermicelli), high-energy and low energy beverages (cola with sugar, carbonated beverages with sugar, fruit punch drinks), and tomatoes as pro-inflammatory group and tea, coffee, dark yellow vegetables (carrots, or squash), leafy green vegetables (cabbage, spinach, or lettuce), snacks (cracker, or potato chips), fruit juice (apple juice, cantaloupe juice, orange juice, or other fruit juice), and pizza anti-inflammatory group. The average daily intakes of each item were multiplied by the proposed weights, and then all the weighted values were summed. Finally, the summed scores were divided by 1000 to reduce the magnitude of the scores. A higher score indicates a more pro-inflammatory diet in both dietary inflammatory indices and vice versa.

### Lifestyle-related measurements

Digital scales (model 707, Seca, Hamburg, Germany) were used to measure the participants’ body weight to the nearest 100 g with light clothes and without shoes. A stadiometer (model 208 Portable Body Meter Measuring Device; Seca) was used to measure height to the nearest 0.5 cm standing while the individuals were barefoot. Body mass index (BMI) was calculated as weight (kg) divided by height (m^2^). As previously reported [30], waist circumference (WC) was measured to the nearest 0.1 cm using a non-elastic tape meter, between the lowest chest ribs and the iliac crest at the umbilicus level, over light clothing, without any pressure on the body skin. Hip circumference (HC) was measured to the nearest 0.1 cm using a non-elastic tape meter, around the widest portion of the buttocks, with the tape parallel to the floor. Waist-to-hip ratio (WHR) was computed as WC (in cm) divided by HC (in cm) [30]. Through face-to-face interviews, physical activity levels were measured using the international physical activity questionnaire (IPAQ) [31]. All results of the IPAQ were expressed as Metabolic Equivalents per week (METs/week) [32, 33].

### Assessment of other variables

Information on age, sex, marital status, socioeconomic status (SES), and current smoking status was obtained using a demographic questionnaire [34]. In the present study, smoking was classified into yes/no groups; ‘yes’ defined subjects who smoked cigarettes as daily or occasionally or ex-smokers, and ‘no’ described the individuals who are non-smokers [35]. SES score was calculated based on three variables, including family size (≤ 4, > 4 people), education levels (academic and non-academic education), and acquisition (house ownership or not). For each of these variables, participants were given a score of 1 (if their family members were ≤ 4, were academically educated or owned a house) or given a score of 0 (if their family members were > 4, or had non-academic education, or leasehold property). Then, the total SES score was computed by summing up the assigned scores (minimum SES score of 0 to a maximum score of 3). An SES score of 3 equated to high, 2 was moderate, and 1 or 0 was low.

### Statistical analysis

Statistical analysis was conducted using Statistical Package Software for Social Science, version 21 (SPSS Inc., Chicago, IL, USA). The normality of variables was examined using the Kolmogorov–Smirnov test and histogram chart. Baseline characteristics and dietary intakes were expressed as mean ± SD or median (25–75 interquartile range) for quantitative variables and numbers and percentages for qualitative variables. Independent sample t-test, Mann–Whitney test, and chi-square were used for testing the differences between cases and controls for normally distributed variables, skewed variables, and categorical variables, respectively. Participants were categorized into tertiles based on the DIS and EDIP scores. The general and dietary data were reported across total DIS and EDIP scores tertiles. *P* for the trend of continuous and categorical variables across DIS and EDIP scores tertiles was assessed using linear regression and chi-square test. The association between DIS and EDIP scores with the odds of NAFLD was assessed using logistic regression. The analysis was adjusted for potential confounders, including age and sex, BMI, physical activity, smoking, SES, waist-to-hip ratio (WHR), and dietary intake of energy. The odds ratio (OR) with 95% confidence interval (CI) of NAFLD across tertiles of DIS and EDIP scores were reported, and *P* values < 0.05 were considered statistically significant.

## Results

The mean (SD) age and BMI of the study population (53% male) were 38.1 (8.8) years and 26.8 (4.3) kg/m^2^, respectively. The median (IQR) of EDIP and DIS were 0.52 (0.34, 0.73), and 0.04 (− 0.55, 0.59), respectively. Our results reported that participants in the cases group had a higher BMI than the individuals in the control group, whereas the mean age and percentage of gender distribution were not different between the cases and the control group. Also, our findings revealed that the median (IQR) of EDIP and DIS of individuals in the case group was higher than subjects in the control group.

General characteristics, nutrient intakes, and DIS components intakes for the study population across tertiles of DIS are presented in Table 1. Participants in the highest tertile of DIS were significantly younger (T1: 40.0 ± 9.9, T2: 38.3 ± 8.2, and T3: 36.6 ± 8.3 years), had higher WC (T1: 88.8 ± 10.5, T2: 90.9 ± 10.3, and T3: 92.0 ± 11.6 cm), WHR (T1: 0.90 ± 0.08, T2: 0.91 ± 0.08, and T3: 0.92 ± 0.08), energy intakes (T1: 2242 ± 624, T2: 2158 ± 593, and T3: 2399 ± 672 kcal/d), and had lower intakes of protein (% of energy) (T1: 14.0 ± 2.7, T2: 13.8 ± 2.4, and T3: 13.1 ± 2.0) and fats (% of energy) (T1: 32.0 ± 6.6, T2: 32.5 ± 7.0, and T3: 30.5 ± 7.0) than subjects in the lowest tertile of DIS (*P* < 0.05).

**Table 1:** General characteristics and dietary intake among study participants based on tertiles of the DIS score among participants

Variables	Tertiles of DIS			*P*-trend
	T1 (n = 191)	T1 (n = 225)	T1 (n = 259)	
Age(year)	40.0 ± 9.9	38.3 ± 8.2	36.6 ± 8.3	< 0.001
Male, n (%)	91 (47.4)	120 (53.6)	147 (56.8)	0.141
Waist circumference (cm)	88.8 ± 10.5	90.9 ± 10.3	92.0 ± 11.6	0.002
Hip circumference (cm)	98.8 ± 8.2	99.8 ± 7.6	99.7 ± 8.2	0.212
WHR	0.90 ± 0.08	0.91 ± 0.08	0.92 ± 0.08	0.003
BMI (kg/m^2^)	26.6 ± 4.1	27.1 ± 4.0	26.8 ± 4.7	0.711
Smoking, n (%)	5 (2.6)	15 (6.7)	8 (3.1)	0.073
Physical activity (MET/min/week)	1485 ± 995	1436 ± 831	1391 ± 832	0.266
Marital status (married)	159 (82.8)	192 (85.7)	214 (82.6)	0.290
Nutrient intake				
Energy (kcal/d)	2242 ± 624	2158 ± 593	2399 ± 672	0.009
Carbohydrate (% of energy)	57.5 ± 7.3	56.2 ± 7.2	58.2 ± 7.0	0.270
Protein (% of energy)	14.0 ± 2.7	13.8 ± 2.4	13.1 ± 2.0	< 0.001
Fat (% of energy)	32.0 ± 6.6	32.5 ± 7.0	30.5 ± 7.0	0.022
DIS components				
DIS score	− 0.95 (− 1.37–− 0.66)	− 0.06 (− 0.25–0.11)	0.77 (0.49–1.23)	< 0.001
Leafy greens and Cruciferous vegetables (g/d)	17.5 (10.8–33.8)	13.1 (7.4–23.9)	10.4 (6.8–21.4)	< 0.001
Tomatoes (g/d)	169 ± 111	110 ± 75	73 ± 58	< 0.001
Apples and berries (g/d)	106.6 (42.2–161.0)	54.1 (22.9–114.6)	29.2 (16.8–60.1)	< 0.001
Deep yellow or orange Vegetables and fruit (g/d)	58.5 (34.2–106.1)	42.0 (24.1–68.2)	27.6 (16.1–47.2)	< 0.001
Other fruits and real fruit juices (g/d)	357 ± 250	227 ± 159	177 ± 135	< 0.001
Other vegetables (g/d)	166 ± 84	133 ± 66	112 ± 59	< 0.001
Legumes (g/d)	11.3 (6.2–20.5)	12.5 (7.2–21.4)	9.2 (5.5–18.4)	0.088
Fish (g/d)	7.2 (4.1–13.4)	6.7 (3.7–12.8)	6.6 (3.2–12.9)	0.260
Poultry (g/d)	21.4 (12.1–33.9)	21.4 (12.1–33.5)	18.2 (10.7–29.2)	0.097
Red and organ meats (g/d)	41.0 ± 33.6	37.6 ± 25.2	37.7 ± 24.3	0.350
Processed meats (g/d)	1.3 (0.1–4.0)	2.7 (0.4–5.7)	2.7 (0.8–6.7)	< 0.001
Added sugars (g/d)	44.4 (25.5–72.5)	45.3 (27.6–73.7)	45.9 (27.9–84.2)	0.350
High-fat dairy (g/d)	103 (44–233)	93 (35–230)	103 (35–232)	0.939
Low-fat dairy (g/d)	272 ± 170	254 ± 192	219 ± 145	< 0.001
Coffee and tea (g/d)	751 (500–1127)	502 (251–750)	275 (250–508)	< 0.001
Nuts (g/d)	4.0 (1.7–8.8)	4.0 (2.1–7.2)	3.3 (1.6–6.5)	< 0.001
Other fats (g/d)	12.8 (7.3–23.9)	14.9 (8.3–29.6)	19.3 (10.5–31.5)	0.042
Refined grains and Starchy vegetables (g/d)	342 ± 113	411 ± 135	600 ± 230	< 0.001

Data are presented as mean ± standard deviation for normally distributed variables and median (25–75 interquartile range) for skewed variables

DIS: dietary inflammation scores

Also, Table 1 showed that the intakes of leafy greens and cruciferous vegetables [T1: 17.5 (10.8–33.8), T2:13.1 (7.4–23.9), and T3: 10.4 (6.8–21.4) g/d], deep yellow or orange vegetables and fruit [T1: 58.5 (34.2–106.1), T2: 42.0 (24.1–68.2), and T3: 27.6 (16.1–47.2) g/d], apples and berries [T1: 106.6 (42.2–161.0), T2: 54.1 (22.9–114.6), and T3: 29.2 (16.8–60.1) g/d], tomatoes (T1: 169 ± 111, T2: 110 ± 75, and T3: 73 ± 58 g/d), other fruits and real fruit juices (T1: 357 ± 250, T2: 227 ± 159, and T3: 177 ± 135 g/d), other vegetables (T1: 166 ± 84, T2: 133 ± 66, and T3: 112 ± 59 g/d), nuts [T1: 4.0 (1.7–8.8), T2: 4.0 (2.1–7.2), and T3: 3.3 (1.6–6.5) g/d], low-fat dairy (T1: 272 ± 170, T2: 254 ± 192, and T3: 219 ± 145 g/d), and coffee and tea [T1: 751 (500–1127), T2: 502 (251–750), and T3: 275 (250–508) g/d] were significantly reduced across DIS score tertile (*P* < 0.05). However, individuals in the highest tertile of the DIS score had higher intakes of processed meat [T1: 1.3 (0.1–4.0), T2: 2.7 (0.4–5.7), and T3: 2.7 (0.8–6.7) g/d] and refined grains and starchy vegetables (T1: 342 ± 113, T2: 411 ± 135, and T3: 600 ± 230 g/d) compared to those in the lowest tertile of DIS (*P* < 0.05).

General characteristics, nutrient intakes, and EDIP components intakes of individuals across tertiles of EDIP are shown in Table 2. Our findings indicate no significant difference in mean age (T1: 38.4 ± 8.9, T2: 37.3 ± 8.6, and T3: 38.7 ± 9.0 years), physical activity (T1: 1530 ± 882, T2: 1376 ± 847, and T3: 1400 ± 906 MET/min/week), BMI (T1: 26.5 ± 4.2, T2: 26.9 ± 3.9, and T3: 27.1 ± 4.7 kg/m2), WC (T1: 89.7 ± 10.2, T2: 90.7 ± 10.7, and T3: 91.6 ± 11.7 cm), WHR (T1: 0.91 ± 0.07, T2: 0.91 ± 0.08, and T3: 0.91 ± 0.08) and % of smoking (T1: 4.7, T2: 4.5, and T3: 3.3%) and married subjects (T1: 82.9, T2: 82.7, and T3: 85.2%) across tertiles of EDIP. However, participants in the highest tertile of EDIP had higher energy intakes (T1: 2089 ± 614, T2: 2222 ± 573, and T3: 2482 ± 663 kcal) and lower intakes of fats (% of energy) (T1: 32.1 ± 6.4, T2: 32.1 ± 7.3, and T3: 30.7 ± 7.1%) than those in the lowest EDIP (*P* < 0.05).

**Table 2:** General characteristics and dietary intake among study participants based on tertiles of the EDIP score among participants

Variables	Tertiles of EDIP			*P*-trend
	T1 (n = 211)	T1 (n = 220)	T1 (n = 244)	
Age (year)	38.4 ± 8.9	37.3 ± 8.6	38.7 ± 9.0	0.618
Male, n (%)	117 (55.5)	104 (47.3)	137 (56.1)	0.112
Waist circumference (cm)	89.7 ± 10.2	90.7 ± 10.7	91.6 ± 11.7	0.064
Hip circumference (cm)	98.9 ± 8.1	99.3 ± 6.8	100.2 ± 8.9	0.077
WHR	0.91 ± 0.07	0.91 ± 0.08	0.91 ± 0.08	0.384
BMI (kg/m^2^)	26.5 ± 4.2	26.9 ± 3.9	27.1 ± 4.7	0.198
Smoking, n (%)	10 (4.7)	10 (4.5)	8 (3.3)	0.684
Physical activity (MET/min/week)	1530 ± 882	1376 ± 847	1400 ± 906	0.153
Marital status (married)	175 (82.9)	182 (82.7)	208 (85.2)	0.799
Nutrient intake				
Energy (kcal/d)	2089 ± 614	2222 ± 573	2482 ± 663	< 0.001
Carbohydrate (% of energy)	56.7 ± 6.7	57.2 ± 7.6	58.0 ± 7.1	0.064
Protein (% of energy)	13.7 ± 2.7	13.4 ± 2.2	13.6 ± 2.2	0.657
Fat (% of energy)	32.1 ± 6.4	32.1 ± 7.3	30.7 ± 7.1	0.033
EDIP components				
EDIP score	0.25 (0.17–0.33)	0.51 (0.45–0.57)	0.83 (0.70–1.0)	< 0.001
Processed meat (serving/week)	0.07 (0.00–0.21)	0.14 (0.00–0.35)	0.14 (0.00–0.35)	< 0.001
Red meat (serving/week)	0.63 (0.35–1.19)	0.77 (0.49–1.33)	0.84 (0.49–1.40)	0.017
Organ meat (serving/week)	0.04 (0.01–0.10)	0.03 (0.01–0.08)	0.03 (0.01–0.09)	0.727
Other fish (serving/week)	0.35 (0.21–0.63)	0.42 (0.21–0.77)	0.44 (0.21–0.91)	0.002
Other vegetables (serving/d)	1.3 ± 0.7	1.9 ± 1.0	2.7 ± 1.4	< 0.001
Refined grains (serving/d)	2.2 ± 1.0	3.2 ± 1.5	5.6 ± 3.3	< 0.001
High-energy beverages (serving/week)	0.21 (0.02–0.42)	0.12 (0.07–0.56)	0.42 (0.03–0.84)	0.012
Tomatoes (serving/d)	0.47 ± 0.39	0.86 ± 0.59	1.30 ± 0.86	< 0.001
Tea (serving/d)	2.4 (1.0–4.2)	2.2 (1.0–3.1)	2.1 (1.0–3.1)	< 0.001
Coffee (serving/week)	0.06 (0.02–0.35)	0.04 (0.01–0.24)	0.02 (0.01–0.21)	0.391
Dark yellow vegetables (serving/d)	0.14 (0.05–0.30)	0.11 (0.05–0.26)	0.10 (0.05–0.24)	0.067
Leafy green vegetables (serving/d)	0.23 (0.13–0.44)	0.24 (0.15–0.47)	0.25 (0.13–0.51)	0.817
Snacks (serving/d)	0.08 (0.01–0.16)	0.09 (0.02–0.19)	0.13 (0.02–0.20)	0.451
Fruit juice (serving/d)	0.21 (0.07–0.27)	0.28 (0.14–0.70)	0.21 (0.07–0.56)	0.449

Data are presented as mean ± standard deviation for normally distributed variables and median (25–75 interquartile range) for skewed variables

EDIP: empirical dietary inflammatory patterns

Also, Table 2 indicated that the intakes of red meat [T1: 0.63 (0.35–1.19), T2: 0.77 (0.49–1.33), and T3: 0.84 (0.49–1.40) serving/week], processed meat [T1: 0.07 (0.00–0.21), T2: 0.14 (0.00–0.35), and T3: 0.14 (0.00–0.35) serving/week], refined grain (T1: 2.2 ± 1.0, T2: 3.2 ± 1.5, and T3: 5.6 ± 3.3 serving/d), high-energy beverages [T1: 0.21 (0.02–0.42), T2: 0.12 (0.07–0.56), and T3: 0.42 (0.03–0.84) serving/week], other vegetables (T1: 1.3 ± 0.7, T2: 1.9 ± 1.0, and T3: 2.7 ± 1.4 serving/d), and tomatoes (T1: 0.47 ± 0.39, T2: 0.86 ± 0.59, and T3: 1.30 ± 0.86 serving/d) were significantly increased across tertiles of EDIP score (*P* < 0.05). However, individuals in the highest tertile of the EDIP score had lower tea intakes [T1: 2.4 (1.0–4.2), T2: 2.2 (1.0–3.1), and T3: 2.1 (1.0–3.1) serving/d) than those in the lowest tertile of EDIP (*P* < 0.05).

The association between the higher scores of EDIP and DIS and the risk of NAFLD is reported in Table 3. In the age and sex-adjusted model, individuals in the second tertile (OR = 1.91; 95% CI 1.22–2.98) and third tertile (OR = 2.83; 95% CI 1.84–4.37) DIS score had higher odds of NAFLD compared to first tertile of DIS (as the reference group) (*P* for trend < 0.001). Also, in the multivariable-adjusted model, after additional adjusting for physical activity, smoking, marital status, WHR, and dietary energy intake, there is a significant positive relationship between DIS score and odds of NAFLD [(OR T2 vs. T1 = 2.01, 95% CI 1.07–3.76) and (OR T3 vs. T1 = 2.54, 95% CI 1.39–4.63)], (P_trend_ = 0.003).

**Table 3:** Odds ratios (ORs) and 95% confidence intervals (CIs) for NAFLD based on tertiles of DIS and EDIP

	Tertiles of scores			*P* for trend
	T1	T2	T3	
DIS				
Median score	− 0.95	− 0.05	0.77	
NAFLD/control	41/150	75/150	109/150	
Crude model	1.00 (Ref)	1.85 (1.19–2.89)	2.68 (1.75–4.09)	< 0.001
Model 1^a^	1.00 (Ref)	1.91 (1.22–2.98)	2.83 (1.84–4.37)	< 0.001
Model 2^b^	1.00 (Ref)	2.01 (1.07–3.76)	2.54 (1.39–4.63)	0.003
EDIP				
Median score	0.25	0.50	0.83	
NAFLD /control	61/150	70/150	94/150	
Crude model	1.00 (Ref)	1.15 (0.76–1.73)	1.54 (1.04–2.29)	0.027
Model 1^a^	1.00 (Ref)	1.17 (0.78–1.78)	1.54 (1.04–2.28)	0.029
Model 2^b^	1.00 (Ref)	0.88 (0.50–1.57)	1.82 (1.02–3.23)	0.031

^a^Adjusted for age and sex

^b^Adjusted for model 1 and physical activity, smoking, marital status, WHR, and dietary intake of energy

According to Table 3, based on the age and sex-adjusted model, individuals in the second tertile of EDIP score did not have higher odds of NAFLD compared to the first tertile of EDIP (OR = 1.17; 95% CI 0.78–1.78), however, participants in the highest tertile of EDIP (third tertile) had higher odds of NAFLD compared to lowest tertile (OR = 1.54; 95% CI 1.04–2.28, P_trend_ = 0.029). In the final model, although the odds of NAFLD in the second tertile of EDIP were not different compared to those in the first tertile of EDIP (OR = 0.88; 95% CI 0.50–1.57), a significantly higher odds of NAFLD was observed in the third tertile of EDIP compared those in the lowest tertile of EDIP (OR = 1.82; 95% CI 1.02–2.23, P_trend_ = 0.031).

## Discussion

In this case–control study, two inflammatory indices, including EDIP and DIS, were used to determine the possible linkage between the inflammatory potential of dietary pattern with the risk of NAFLD among Tehranian adults. Our results showed that higher adherence to a pro-inflammatory dietary pattern, characterized by higher EDIP and DIS scores, was associated with a higher adds of NAFLD, independent of confounding factors, including age, sex, physical activity, smoking, marital status, WHR, and dietary energy intake.

The current study results are in agreement with the findings of limited studies that assessed the possible association of DIS and EDIP with the risk of NAFLD [25, 26]. A prospective cohort study suggested that a diet with high inflammatory potential may increase NAFLD risk [25]. Also, the nested case–control study revealed that individuals with a higher score of DIS may be more prone to increased odds of metabolic-associated fatty liver disease [26]. Furthermore, the current study results are comparable with the results of two studies that examined the association of DII with the risk of NAFLD and reported a positive association between a higher degree of liver damage [19] and increased risk of NAFLD [18]. Generally, it can be said that the results of our study are somewhat in agreement with the results of the studies mentioned above, most of which have emphasized the potential pro-inflammatory role of a dietary pattern with a higher score of EDIP or DIS in increment the risk of NAFLD.

Several epidemiological studies previously reported that higher scores of EDIP or DIS might be related to an increased risk of cardiometabolic disorders such as diabetes [23], metabolic syndrome [16, 36], dyslipidemia [37], and central adiposity [36, 37]. A cohort study on postmenopausal women reported that high inflammatory potentials of the diet, determined by EDIP score, may increase the risk of diabetes [23]. The Shakeri Z et al. study indicated that a higher score of EDIP can be the leading risk factor for the development of MetS and its components such as dysglycemia, dyslipidemia, and central obesity [36]. Also, a cross-sectional study reported that higher EDIP scores have led to increased odds of unhealthy metabolic phenotype, hyperglycemia, low-HDL-C, and higher WC in obese people [37]. These studies suggested that a higher score of EDIP is associated with greater odds of metabolic disorders that somehow play a role in the onset or progression of fatty liver. Furthermore, in two cohort studies, the possible pro-inflammatory effect of diet, determined by higher DIS score, in the pathogenesis of chronic metabolic diseases have also been investigated [16, 38]. A population-based cohort study on Iranian adults revealed that a higher score of DIS might be linked with a higher risk of MetS [16], but no significant association was observed between a higher score of DIS with increased T2D incidence in another cohort study [38].

NAFLD is a low-grade inflammatory chronic disease that the process of its occurrence and progression is accelerated by the high level of pro-inflammatory cytokines, including Interleukin‐1, TNF‐α, and Interleukin‐6 and the high level secretion of adipokines. These inflammatory factors trigger various processes, including lipotoxicity, hepatocyte cell death, liver inflammation, fibrosis, and pathological angiogenesis [39]. Therefore, any environmental factor affecting the creation or exacerbation of inflammatory conditions, such as greater adherence to a pro-inflammatory dietary pattern, which is associated with increased cellular or serum levels of these inflammatory markers, can increase the risk of fatty liver disease or its progression. The findings of our study can be justified by these proposed mechanisms because, we showed that a dietary pattern with higher scores of DIS and EDIP is rich in pro-inflammatory food components, including red and processed meat, simple sugar, refined grains, starchy vegetables, and saturated fats and poor in anti-inflammatory food items including fruits, leafy green vegetables, legumes, nuts, dairy products, and fish that may contribute to the pathogenesis of liver dysfunction development and increased risk of NAFLD via provoking systemic inflammation markers production. In other words, the cumulative and combined effect of the pro-inflammatory food components [40], along with low consumption of anti-inflammatory foods in the form of dietary inflammatory indices called DIS and EDIP, can cause the accumulation of fat in liver cells, lipotoxicity, and hepatocyte cell dysfunction through the inflammatory pathway and consequently accelerate the NAFLD progression. Also, according to DIS and EDIP, these dietary scores are poor in antioxidant vitamins and minerals and phenolic compounds and have a high saturated fatty acid content that mediates its effects on hepatic metabolic abnormality through possible mechanisms including inflammatory and oxidant properties and increasing visceral adiposity.

Therefore, based on the available evidence and the findings of this study, a higher adherence to a diet with the lowest DIS or EDIP score can be helpful in the prevention of metabolic disorders such as NAFLD. Because such a diet is a healthy diet with anti-inflammatory characteristics, which emphasizes increasing intakes of vegetables, fruits, legumes, whole grain, nuts, and dairy products, and lower intakes of simple sugar, red and processed meat, and saturated fat, may be inversely associated with risk of metabolic disorders such as NAFLD [41, 42] via reducing the production of pro-inflammatory indicators such as hs-CRP, ILs, and TNF-a [43–45].

Our study had several strengths. The current study is the first study that examined the association of pro-inflammatory dietary exposures, determined by EDIP and DIS scores, with the risk of NAFLD in adults. Also, a validated and reproducible FFQ was used to collect individuals’ dietary intakes, which expert dietitians completed in a face-to-face interview; this questionnaire was not a self-reported questionnaire that minimizes measurement bias. Furthermore, a validated questionnaire was used to collect data on the physical activity levels of individuals. Despite the above-mentioned strengths, the limitations of our study should be mentioned. First, this study's case–control design led us not to discover the causality between exposures and outcomes. Second, although a biopsy of the liver and magnetic resonance imaging (MRI) technique is the gold standard and more accurate tests for diagnosis of NAFLD, we used ultrasonography test to diagnose NAFLD in participants; it is worth mentioning that because of the limitations and complications of biopsy and high cost and low availability of MRI, using noninvasive methods such as ultrasonography is applicable and reliable to diagnosis NAFLD in clinical practice [46]. Third, we could not specify the individuals who ever smoked, which could be more helpful for controlling the impact of smoking as a confounding factor in the analysis of the current study. Furthermore, some inherent limitations of case–control studies, including selection bias and information bias in determining exposure or outcome, should be considered in interpreting the results. Finally, despite the adjustment of various potential confounders, our study design cannot eliminate all possible confounders, and the effects of some residual confounders may have occurred.

## Conclusion

In conclusion, our case–control study suggested that higher scores of EDIP and DIS are related to an increased odds of NAFLD in Iranian adults. Therefore, our findings showed that a higher ratio of pro- to anti-inflammatory dietary exposures may increase the risk of hepatic abnormalities.

## Data Availability

The datasets analyzed in the current study are available from the corresponding author on reasonable request.

## Abbreviations

BMI: Body mass index
CI: Confidence interval
CRP: C-reactive protein
CVDs: Cardiovascular diseases
DII: Dietary inflammatory index
DIS: Dietary inflammation scores
EDIP: Empirical dietary inflammatory pattern
FCT: Food Composition Table
FFQ: Food frequency questionnaire
ILs: Interleukins
IPAQ: International physical activity questionnaire
OR: Odds ratio
METs: Metabolic Equivalents
MRI: Magnetic resonance imaging
NAFLD: Non-alcoholic fatty liver disease
NASH: Non-alcoholic steatohepatitis
T2D: Type 2 diabetes
USG: Ultrasonography scan
USDA: US Department of Agriculture
SES: Socioeconomic status
SPSS: Statistical Package Software for Social Science
WC: Waist circumference
WHR: Waist-to-hip ratio
